# Regional and patient characteristic disparities in the outcomes of minimally invasive surgery for colorectal cancer in Japan

**DOI:** 10.1002/ags3.70007

**Published:** 2025-04-03

**Authors:** Atsushi Hamabe, Arata Takahashi, Hiraku Kumamaru, Hiroshi Hasegawa, Koki Otsuka, Yoshihiro Kakeji, Ken Shirabe, Masafumi Inomata, Yuko Kitagawa, Ichiro Takemasa

**Affiliations:** ^1^ Department of Surgery, Surgical Oncology and Science Sapporo Medical University Sapporo Japan; ^2^ Department of Gastroenterological Surgery, Graduate School of Medicine Osaka University Osaka Japan; ^3^ Department of Health Policy and Management, School of Medicine Keio University Tokyo Japan; ^4^ Department of Healthcare Quality Assessment, Graduate School of Medicine The University of Tokyo Tokyo Japan; ^5^ Division of Gastrointestinal Surgery, Department of Surgery, Graduate School of Medicine Kobe University Kobe Japan; ^6^ Project Management Subcommittee, The Japanese Society of Gastroenterological Surgery Tokyo Japan; ^7^ Department of Advanced Robotic and Endoscopic Surgery Fujita Health University School of Medicine Toyoake Japan; ^8^ Database Committee, The Japanese Society of Gastroenterological Surgery Tokyo Japan; ^9^ Department of General Surgical Science Gunma University Graduate School of Medicine Maebashi Gunma Japan; ^10^ The Japanese Society of Gastroenterological Surgery Tokyo Japan; ^11^ Department of Gastroenterological and Pediatric Surgery, Faculty of Medicine Oita University Oita Japan; ^12^ Academic Committee of Japan Society for Endoscopic Surgery Tokyo Japan; ^13^ Department of Surgery Keio University School of Medicine Tokyo Japan; ^14^ Japan Society for Endoscopic Surgery Tokyo Japan; ^15^ Department of Gastroenterological Surgery Osaka International Medical and Science Center, Osaka Keisatsu Hospital Osaka Japan

**Keywords:** colorectal neoplasms, minimally invasive surgical procedures, National Clinical Database

## Abstract

**Aim:**

The use of minimally invasive surgery, including laparoscopic and robotic surgery, for gastrointestinal cancer has been rapidly increasing. This study aimed to clarify whether differences in minimally invasive surgery outcomes are associated with regional and patient characteristics.

**Methods:**

A total of 123 771 right hemicolectomy and 126 965 low anterior resection cases performed between 2013 and 2019 were selected from the National Clinical Database for analysis. Patients were stratified by regional and economic variables, and open and minimally invasive surgical outcomes were evaluated.

**Results:**

In secondary medical regions characterized by urban settings and numerous designated cancer care hospitals, the observed 30‐day mortality of low anterior resections was lower only in the minimally invasive surgery group. For right hemicolectomies in regions with many designated cancer care hospitals, the observed incidence of postoperative complications was also lower in the minimally invasive group. Residents of high‐income areas undergoing low anterior resection had a lower frequency of 30‐day reoperation regardless of the type of surgery and a lower 30‐day mortality in the minimally invasive group. For both right hemicolectomy and low anterior resection, patients with longer travel distances had fewer postoperative complications and lower 30‐day reoperation rates in the minimally invasive group than in the open surgery group.

**Conclusion:**

This study found regional and patient characteristic disparities in minimally invasive surgical outcomes; national policies should be implemented to address these inequities.

## Graphical abstract

This study aimed to clarify whether differences in minimally invasive surgery outcomes are associated with regional and patient characteristics. This study found regional and patient characteristic disparities in minimally invasive surgical outcomes; national policies should be implemented to address these inequities.

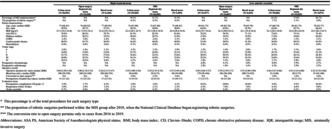

## 1 INTRODUCTION

The proportion of minimally invasive surgeries (MIS) for gastrointestinal cancer has been increasing annually worldwide. According to data from Japan in 2020, 54.2% of right/left hemicolectomies and 72.9% of low anterior resections (LAR) for colorectal cancer were performed laparoscopically.^1^ Similarly, the number of MIS performed for other gastrointestinal diseases is also rising. In Japan, the number of robotic surgeries performed has rapidly increased since robotic surgery for gastrointestinal cancer was covered by insurance in April 2018.

The technology for MIS in gastrointestinal cancer has advanced rapidly, and its diversity is expected to increase with the future introduction of new technologies. With the provision of advancing technologies, patients have more opportunities to benefit from reduced invasiveness, leading to improved quality of life.^2^, ^3^, ^4^, ^5^ However, for surgeons, continuous efforts are required to learn and maintain skills related to specialized equipment and techniques. To this end, urban areas are presumed to be advantageous for the safe dissemination of MIS because of the presence of numerous physicians, organ‐ or disease‐specific medical care, and easily accessible information on advanced technologies. From the patient's perspective, visiting hospitals that have actively introduced MIS may be intentional, with the expectation of less invasiveness and better cosmetic outcomes. However, whether patients can visit such hospitals may depend on their social and economic situation. Clarifying whether MIS outcomes differ based on regional and patient characteristics can facilitate the establishment of effective measures to improve MIS treatment outcomes nationwide.

In this study, we aimed to assess any disparities in the implementation and treatment outcomes of MIS for colorectal cancer based on various regional and economic characteristics, as well as the convenience of access to medical care.

## 2 METHODS

### 2.1 Patients

Of all the types of gastrointestinal surgeries included in the National Clinical Database (NCD), the Japanese Society of Gastroenterological Surgery has designated nine procedures as targets for evaluating medical standards under its board certification system because of their importance in assessing healthcare quality. These procedures include esophagectomy, distal gastrectomy, total gastrectomy, right hemicolectomy (RHC), LAR, hepatectomy, pancreatoduodenectomy, acute diffuse peritonitis surgery, and liver transplantation.^6^ Of these, RHC and LAR were selected for this study because of the high frequency and penetration rate of MIS.

Using data from the NCD, we analyzed RHC and LAR cases from 2013 to 2019, setting this range to ensure sufficient numbers of clinical data entry items and to avoid the impact of the COVID‐19 pandemic. Emergency surgeries and cases involving simultaneous surgeries performed in different regions were excluded. A total of 123 771 RHC and 126 965 LAR cases were classified into open surgery and MIS groups, with MIS procedures defined as laparoscopic and robotic surgeries.

The study protocol was approved by the Institutional Review Board of Sapporo Medical University. Individual written informed consent was waived because of the retrospective design.

### 2.2 Medical regions

In Japan, multiple medical regions are designated under the national system, and medical plans are established for each region. These include primary medical regions (generally at the municipal level), secondary medical regions that generally consist of multiple municipalities, and tertiary medical regions at the prefectural level. Based on Japan's 7th Medical Plan, there are 335 secondary medical regions in Japan, each with designated numbers of doctors, nurses, and hospital beds.^7^ In this study, we classified the secondary medical regions using multiple indicators and evaluated the characteristics in open surgery and MIS treatment outcomes for each secondary medical region.

### 2.3 Indicators for classifying secondary medical regions

#### 2.3.1 Regional classification

Following previous reports, secondary medical regions were classified into three types: (1) urban areas with a population over 1 million or a population density >2000 people/km^2^; (2) regional city areas with a population >200 000 or a population of 100 000–200 000 and a population density >200 people/km^2^; and (3) rural areas not falling into the urban or regional city categories.^8^

Population and population density data for the secondary medical regions were obtained from information published in 2014.^9^ We evaluated the outcomes in 49 urban, 157 regional city, and 129 rural secondary medical regions.

#### 2.3.2 Physician distribution index

To objectively assess the nationwide distribution of physicians, the Japanese government has provided a physician distribution index that considers regional medical needs, population composition, and the sex and age of physicians, as follows:

$$\text{Physician distribution index}=\frac{\text{Standardized number of physicians}}{\left(\text{Regional population}÷100,000\right)\times \text{Regional standardized treatment rate ratio}}$$

$$\begin{array}{cc} \text{Standardized number of physicians}= & \sum \left(\text{Number of physicians}\;\mathrm{by}\;\mathrm{sex}\;\text{and}\;\mathrm{age}\;\text{group}\times \text{Average working hours}\;\mathrm{by}\;\mathrm{sex}\;\text{and}\;\mathrm{age}\;\text{group}\right)\\ & ÷\text{Average working hours of}\;\mathrm{all}\;\text{physicians} \end{array}$$

$$\text{Regional standardized treatment rate ratio}=\frac{\text{Expected regional treatment rate}}{\text{National expected treatment rate}}$$

$$\text{Expected regional treatment rate}=\frac{\sum \left(\text{National treatment rate}\;\mathrm{by}\;\mathrm{sex}\;\text{and}\;\mathrm{age}\;\text{group}\times \text{Regional population}\;\mathrm{by}\;\mathrm{sex}\;\text{and}\;\mathrm{age}\;\text{group}\right)}{\text{Regional population}}$$

Secondary medical areas were divided into three groups (upper, middle, and lower) based on the physician distribution index published in 2019.^7^

#### 2.3.3 Designated cancer care hospital

Designated cancer care hospitals have been established in Japan to ensure high‐quality cancer care nationwide. Based on the number of designated cancer care hospitals in each secondary medical region as of 2021, the regions were classified into those with ≥4 (*n* = 18), 1–3 (*n* = 254), and no hospitals (*n* = 63).^10^

### 2.4 Indicators for classifying patient characteristics

#### 2.4.1 Income

The assumed income for each patient was determined based on their residence (specifically, the postal code) and the taxable income by municipality published by the Ministry of Internal Affairs and Communications.^11^ Income was based on the average value in 2013 and divided into quartiles.

#### 2.4.2 Travel distance to hospital

Latitude and longitude information were assigned based on the patient's residence and the medical institution's location to calculate the travel distance via road networks and travel time by car between the two points. ArcGIS (Esri, Redlands, CA, United States) software was used for this analysis. Travel time and distance were calculated using the 2016 Esri road network data.^12^ Travel distance was divided into quartiles from the shortest to the longest.

### 2.5 Outcomes

Following previous NCD survey reports, the evaluation items included the incidence of postoperative complications classified as Clavien–Dindo (CD) Grade ≥3, reoperation rate within 30 days postoperatively, and 30‐day postoperative mortality rate.^1^ In order to evaluate the characteristics of the surgeries potentially associated with the above regional and patient factors, we used the following characteristics: patients' age, sex, body mass index (BMI), ASA performance status (PS), comorbidities (presence of diabetes, chronic obstructive pulmonary disease, cardiovascular complications, and dialysis dependency), preoperative chemotherapy and radiotherapy, tumor stage, operative time, blood loss, conversion to open surgery, and postoperative hospital stay. Cardiovascular complications were defined as the presence of any of the following: hypertension, congestive heart failure, a history of myocardial infarction, angina, previous percutaneous coronary intervention, prior cardiac surgery, a history of surgery related to symptoms of arterial occlusive disease, or symptoms of arterial occlusive disease.

### 2.6 Statistical analysis

We evaluated the frequencies of MIS based on the regional and patient factors described above for RHC and LAR. Then, we evaluated the frequencies and categorizations of the patient and surgical characteristics as well as the treatment outcomes separately for open surgery and MIS. No statistical tests were conducted as these assessments were purely descriptive.

## 3 RESULTS

### 3.1 Regional classification

The results based on regional classification are summarized in Table 1. For RHC, the proportion of MIS cases was highest in urban areas (59.1%), followed by regional cities (51.7%) and rural areas (41.5%). The conversion rate to open surgery was highest in rural areas (3.6%), followed by urban areas (2.8%) and regional cities (2.5%). The incidence of CD grade ≥3 complications or reoperation rates in each group was approximately 6.0% and 3.0%, respectively; however, the 30‐day postoperative mortality rate was lower in urban areas (0.7%) than in rural areas (1.0%). For MIS RHC, no differences in postoperative complications or reoperation rates were found; however, the 30‐day mortality rate was twice that of rural areas (0.4%) than in urban areas (0.2%) and regional cities (0.2%).

**TABLE 1:** Regional classification.

	Right hemicolectomy						Low anterior resection					
	Open surgery			MIS			Open surgery			MIS		
	Urban areas (*N* = 26 591)	Regional city areas (*N* = 31 410)	Rural areas (*N* = 6444)	Urban areas (*N* = 29 205)	Regional city areas (*N* = 26 302)	Rural areas (*N* = 3819)	Urban areas (*N* = 21 077)	Regional city areas (*N* = 24 467)	Rural areas (*N* = 4419)	Urban areas (*N* = 39 604)	Regional city areas (*N* = 33 525)	Rural areas (*N* = 3873)
Percentage of MIS implementation^a^	NA	NA	NA	59.1%	51.7%	41.5%	NA	NA	NA	65.3%	57.8%	46.7%
The proportion of robotic surgery^b^	NA	NA	NA	0.2%	0.2%	0.4%	NA	NA	NA	7.7%	5.1%	2.0%
Patient characteristics												
Age, years, median (IQR)	75 (68–81)	76 (68–82)	77 (69–83)	74 (67–80)	74 (67–80)	75 (68–81)	69 (61–75)	69 (62–76)	70 (63–78)	67 (60–74)	68 (61–75)	70 (63–77)
Sex, male	49.9%	48.7%	47.5%	50.2%	50.0%	50.0%	64.8%	65.0%	65.9%	65.0%	65.7%	66.6%
BMI (kg/m^2^)	21.5 (19.2–24.0)	21.7 (19.4–24.1)	21.6 (19.3–24.1)	22.4 (20.1–24.7)	22.5 (20.2–24.9)	22.6 (20.4–24.8)	22.0 (19.7–24.4)	22.2 (20.0–24.5)	22.1 (19.8–24.4)	22.5 (20.3–24.8)	22.6 (20.4–24.9)	22.5 (20.3–24.7)
ASA PS 2≤	78.8%	80.2%	78.7%	81.5%	82.5%	81.6%	70.2%	70.3%	71.1%	73.6%	75.1%	74.4%
Diabetes mellitus	19.2%	20.1%	19.5%	20.4%	20.7%	21.8%	17.8%	19.2%	19.8%	17.6%	19.0%	20.0%
COPD	3.4%	3.0%	2.4%	3.8%	2.8%	2.1%	3.8%	3.2%	2.9%	4.0%	3.3%	2.1%
Cardiovascular disease	40.9%	45.7%	46.9%	44.4%	48.0%	49.8%	35.4%	38.8%	41.8%	36.8%	40.5%	44.8%
Hemodialysis	0.8%	0.8%	0.6%	0.7%	0.8%	0.5%	0.5%	0.6%	0.5%	0.5%	0.5%	0.6%
Tumor stage												
0	2.0%	1.8%	2.1%	3.2%	3.5%	3.9%	1.5%	1.8%	2.0%	1.7%	2.1%	2.6%
I	14.0%	13.0%	13.6%	25.7%	24.9%	25.1%	23.1%	22.9%	23.0%	34.7%	33.4%	31.2%
II	35.7%	35.5%	37.6%	32.6%	32.3%	33.6%	30.4%	30.5%	30.4%	26.9%	26.1%	28.4%
III	38.0%	39.3%	37.9%	34.1%	34.9%	33.8%	39.2%	39.3%	39.5%	34.0%	35.6%	34.8%
IV	10.3%	10.4%	8.8%	4.5%	4.4%	3.6%	5.8%	5.5%	5.0%	2.7%	2.8%	3.0%
Preoperative chemotherapy	NA	NA	NA	NA	NA	NA	6.8%	6.1%	5.0%	8.0%	5.9%	4.7%
Preoperative radiotherapy	NA	NA	NA	NA	NA	NA	2.8%	1.8%	1.4%	4.2%	2.9%	2.5%
Operative results												
Operative duration (hr: min), median (IQR)	3:04 (2:20–3:58)	3:00 (2:18–3:53)	2:56 (2:15–3:47)	3:43 (3:00–4:36)	3:39 (2:55–4:34)	3:47 (3:03–4:44)	4:05 (3:04–5:27)	3:59 (2:59–5:17)	3:55 (3:00–5:09)	4:46 (3:47–6:06)	4:41 (3:41–6:00)	4:52 (3:52–6:06)
Blood loss (mL), median (IQR)	100 (30–250)	100 (34–240)	100 (40–235)	30 (6–80)	28 (6–75)	40 (10–100)	179 (50–436)	186 (50–420)	200 (74–422)	21 (5–80)	20 (5–75)	35 (10–100)
Conversion to open surgery^c^	NA	NA	NA	2.8%	2.5%	3.6%	NA	NA	NA	1.8%	1.6%	3.6%
Postoperative hospital stay (days), median (IQR)	15 (11–22)	15 (12–22)	17 (13–25)	11 (9–15)	12 (9–15)	13 (10–17)	17 (13–27)	18 (13–28)	19 (14–31)	15 (11–21)	14 (11–21)	16 (12–25)
Evaluation items												
Postoperative complication CD grade ≥3	5.9%	5.7%	6.3%	3.8%	3.6%	4.1%	10.2%	10.0%	10.6%	9.8%	9.5%	10.4%
Reoperation within 30 days	3.0%	2.8%	3.2%	2.1%	2.1%	2.3%	6.5%	7.0%	7.6%	6.4%	6.6%	8.0%
30‐day mortality	0.7%	0.8%	1.0%	0.2%	0.2%	0.4%	0.5%	0.4%	0.5%	0.2%	0.3%	0.5%

Abbreviations: ASA PS, American Society of Anesthesiologists physical status; BMI, body mass index; CD, Clavien–Dindo; COPD, chronic obstructive pulmonary disease; IQR, interquartile range; MIS, minimally invasive surgery.

^a^ This percentage is of the total procedures for each surgery type.

^b^ The proportion of robotic surgeries performed within the MIS group after 2018, when the National Clinical Database began registering robotic surgeries.

^c^ The conversion rate to open surgery pertains only to cases from 2016 to 2019.

For LAR, the percentage of MIS cases was highest in urban areas (65.3%), followed by regional cities (57.8%) and rural areas (46.7%). The proportion of robotic surgery within the MIS group was highest in urban areas (7.7%), followed by regional cities (5.1%) and rural areas (2.0%). The conversion rate to open surgery was highest in rural areas (3.6%), followed by urban areas (1.8%) and regional cities (1.6%). In both the open surgery and MIS groups, the rates for preoperative radiotherapy and chemotherapy were higher in urban areas than in rural ones. For open surgery LAR, the reoperation rate was highest in rural areas (7.6%); however, postoperative complications or mortality rates were similar between the groups. For MIS LAR, the incidence of postoperative complications was approximately 10.0% in each group; however, both the reoperation rate and the 30‐day mortality rate were highest in rural areas (8.0% and 0.5%, respectively).

### 3.2 Physician distribution index

The results stratified by the physician distribution index are detailed in Table 2. For RHC, the percentage of MIS cases was highest in the upper regions (51.8%), followed by the middle (43.5%) and lower regions (30.8%). The conversion rate to open surgery was highest in the lower regions (4.3%), followed by the middle (2.7%) and upper regions (2.6%). For open surgery RHC, the postoperative complications, reoperation rates, and mortality rates were similar between the groups. However, for MIS RHC, the mortality rate in the middle regions was twice that of the upper and lower regions.

**TABLE 2:** Physician distribution index.

	Right hemicolectomy						Low anterior resection					
	Open surgery			MIS			Open surgery			MIS		
	Upper (*N* = 40 210)	Middle (*N* = 16 542)	Lower (*N* = 7693)	Upper (*N* = 43 172)	Middle (*N* = 12 731)	Lower (*N* = 3423)	Upper (*N* = 31 709)	Middle (*N* = 12 440)	Lower (*N* = 5814)	Upper (*N* = 57 214)	Middle (*N* = 15 884)	Lower (*N* = 3904)
Percentage of MIS implementation^a^	NA	NA	NA	51.8%	43.5%	30.8%	NA	NA	NA	64.3%	56.1%	40.2%
The proportion of robotic surgery^b^	NA	NA	NA	0.2%	0.2%	0.2%	NA	NA	NA	7.6%	2.9%	1.4%
Patient characteristics												
Age, years, median (IQR)	75 (68–82)	75 (68–82)	76 (69–83)	74 (67–80)	74 (68–80)	75 (68–81)	69 (61–76)	70 (63–77)	69 (63–77)	67 (60–74)	68 (61–75)	69 (63–76)
Sex, male	48.9%	49.8%	48.1%	49.9%	50.6%	50.7%	64.3%	65.8%	66.8%	65.1%	66.1%	66.3%
BMI (kg/m^2^)	21.6 (19.3–24.0)	21.6 (19.3–24.0)	21.8 (19.5–24.3)	22.4 (20.1–24.8)	22.5 (20.3–24.8)	22.7 (20.4–25.0)	22.0 (19.8–24.4)	22.1 (19.8–24.5)	22.3 (20.1–24.6)	22.5 (20.3–24.8)	22.6 (20.4–24.8)	22.6 (20.4–24.8)
ASA PS 2≤	80.1%	79.1%	77.1%	82.2%	81.5%	80.0%	70.0%	71.2%	70.3%	74.5%	73.3%	74.9%
Diabetes mellitus	19.4%	20.3%	19.6%	20.5%	21.3%	20.5%	18.3%	19.0%	19.7%	18.1%	18.7%	19.9%
COPD	3.2%	3.1%	2.4%	3.3%	3.4%	1.8%	3.4%	3.9%	2.4%	3.7%	3.4%	2.6%
Cardiovascular disease	43.0%	44.5%	46.6%	45.5%	48.1%	49.9%	36.5%	38.9%	41.3%	38.1%	40.2%	43.5%
Hemodialysis	0.8%	0.8%	0.6%	0.8%	0.7%	0.4%	0.5%	0.6%	0.4%	0.5%	0.5%	0.6%
Tumor stage												
0	1.9%	2.0%	1.9%	3.2%	3.7%	3.9%	1.6%	1.8%	2.1%	1.8%	2.2%	2.7%
I	13.9%	12.5%	13.4%	25.3%	24.8%	27.1%	23.3%	22.0%	23.2%	34.4%	32.9%	33.1%
II	35.4%	36.6%	36.2%	32.2%	32.9%	34.4%	30.3%	31.2%	29.7%	26.3%	27.4%	27.6%
III	38.3%	38.7%	39.9%	34.6%	34.7%	31.6%	38.9%	39.7%	40.3%	34.6%	35.1%	34.6%
IV	10.5%	10.2%	8.6%	4.6%	3.9%	3.0%	5.9%	5.2%	4.7%	2.9%	2.3%	2.0%
Preoperative chemotherapy	NA	NA	NA	NA	NA	NA	6.9%	5.6%	4.6%	7.7%	5.0%	3.1%
Preoperative radiotherapy	NA	NA	NA	NA	NA	NA	2.6%	1.7%	1.0%	4.1%	2.1%	1.7%
Operative results												
Operative duration (hr: min), median (IQR)	3:05 (2:22–4:00)	2:55 (2:13–3:47)	2:52 (2:13–3:42)	3:44 (3:01–4:38)	3:36 (2:53–4:29)	3:35 (2:50–4:32)	4:09 (3:07–5:31)	3:52 (2:52–5:08)	3:41 (2:48–4:50)	4:48 (3:48–6:10)	4:35 (3:36–5:48)	4:31 (3:30–5:46)
Blood loss (mL), median (IQR)	100 (30–250)	100 (38–243)	100 (40–238)	30 (6–80)	30 (68–76)	37 (10–100)	178 (50–424)	200 (67–443)	180 (58–400)	20 (5–80)	25 (5–80)	30 (8–94)
Conversion to open surgery^c^	NA	NA	NA	2.6%	2.7%	4.3%	NA	NA	NA	1.6%	2.4%	2.5%
Postoperative hospital stay (days), median (IQR)	15 (11–22)	16 (12–23)	15 (11–23)	12 (9–15)	12 (9–15)	12 (10–16)	18 (13–27)	18 (13–28)	18 (13–28)	15 (11–21)	14 (11–22)	15 (11–23)
Evaluation items												
Postoperative complication CD grade ≥ 3	5.9%	5.7%	5.7%	3.7%	4.0%	4.2%	10.0%	10.6%	9.8%	9.6%	9.9%	9.5%
Reoperation within 30 days	3.0%	2.8%	3.0%	2.1%	2.2%	2.2%	6.6%	7.0%	7.3%	6.6%	6.5%	7.0%
30‐day mortality	0.7%	0.8%	0.8%	0.2%	0.4%	0.2%	0.5%	0.5%	0.5%	0.2%	0.3%	0.3%

Abbreviations: ASA PS, American Society of Anesthesiologists physical status; BMI, body mass index; CD, Clavien–Dindo; COPD, chronic obstructive pulmonary disease; IQR, interquartile range; MIS, minimally invasive surgery.

^a^ This percentage is of the total procedures for each surgery type.

^b^ The proportion of robotic surgeries performed within the MIS group after 2018, when the National Clinical Database began registering robotic surgeries.

^c^ The conversion rate to open surgery pertains only to cases from 2016 to 2019.

For LAR, the proportion of MIS cases was highest in the upper regions (64.3%), followed by the middle (56.1%) and lower regions (40.2%). The proportion of robotic surgery within the MIS group was highest in the upper regions (7.6%). The conversion rate to open surgery was lowest in the upper regions (1.6%), followed by the middle (2.4%) and lower regions (2.5%). The rates for preoperative radiotherapy and chemotherapy were higher in upper regions than in lower regions in both the open surgery and MIS groups. For open surgery LAR, similar to RHC cases, no obvious differences in complications, reoperation rates, or mortality rates were observed between the groups. For MIS LAR, the 30‐day mortality rate was comparable between the groups.

### 3.3 Designated cancer care hospital

The results based on the number of designated cancer care hospitals are presented in Table 3. For RHC, the percentage of MIS cases was highest in regions with ≥4 hospitals (56.0%), followed by regions with 1–3 (45.9%) and no hospitals (33.2%). The conversion rate to open surgery was highest in the regions with no hospitals (6.5%), followed by those with ≥4 hospitals (3.1%) and 1–3 hospitals (2.5%). For open surgery RHC, no significant differences in the incidence of CD grade ≥3 complications, reoperation rates, or mortality rates were found between the groups. For MIS RHC, lower incidence of CD grade ≥3 complications were observed in regions with ≥4 (3.6%) or 1–3 hospitals (3.8%) than in regions with no hospitals (5.3%). Reoperation and mortality rates were similar between the groups.

**TABLE 3:** Designated cancer care hospital.

	Right hemicolectomy						Low anterior resection					
	Open surgery			MIS			Open surgery			MIS		
	≥4 hospitals (*N* = 13 096)	1–3 hospitals (*N* = 48 856)	No hospitals (*N* = 2493)	≥4 hospitals (*N* = 16 681)	1–3 hospitals (*N* = 41 406)	No hospitals (*N* = 1239)	≥4 hospitals (*N* = 10 744)	1–3 hospitals (*N* = 37 468)	No hospitals (*N* = 1751)	≥4 hospitals (*N* = 22 883)	1–3 hospitals (*N* = 52 887)	No hospitals (*N* = 1232)
Percentage of MIS implementation^a^	NA	NA	NA	56.0%	45.9%	33.2%	NA	NA	NA	68.0%	58.5%	41.3%
The proportion of robotic surgery^b^	NA	NA	NA	0.2%	0.2%	0.2%	NA	NA	NA	8.9%	5.2%	0.6%
Patient characteristics												
Age, years, median (IQR)	75 (67–82)	75 (68–82)	77 (69–83)	74 (67–80)	74 (67–80)	75 (68–82)	68 (61–75)	69 (62–76)	71 (64–78)	67 (59–74)	68 (60–75)	71 (63–78)
Sex, male	49.1%	49.1%	48.0%	50.0%	50.1%	51.2%	63.1%	65.5%	66.5%	64.5%	65.7%	66.2%
BMI (kg/m^2^)	21.5 (19.2–24.0)	21.6 (19.4–24.1)	21.6 (19.3–24.1)	22.3 (20.1–24.7)	22.5 (20.2–24.8)	22.4 (20.2–24.8)	21.9 (19.7–24.4)	22.1 (19.9–24.5)	22.2 (19.9–24.4)	22.5 (20.2–24.8)	22.5 (20.4–24.8)	22.7 (20.2–24.6)
ASA PS 2≤	80.1%	79.5%	76.4%	81.8%	82.0%	82.1%	69.2%	70.7%	69.2%	74.3%	74.2%	79.1%
Diabetes mellitus	19.5%	19.7%	19.7%	20.6%	20.7%	21.1%	17.4%	19.0%	18.3%	18.0%	18.4%	20.5%
COPD	3.1%	3.1%	2.4%	2.9%	3.4%	1.9%	2.9%	3.6%	2.7%	3.1%	3.9%	2.3%
Cardiovascular disease	41.7%	44.2%	48.1%	44.0%	47.1%	51.6%	34.2%	38.3%	43.1%	37.0%	39.4%	46.7%
Hemodialysis	0.8%	0.8%	0.7%	0.8%	0.7%	0.3%	0.5%	0.5%	0.6%	0.5%	0.5%	0.5%
Tumor stage												
0	1.6%	2.0%	1.5%	2.7%	3.6%	4.0%	1.7%	1.7%	2.7%	1.6%	2.1%	2.4%
I	13.0%	13.6%	13.5%	25.3%	25.4%	23.5%	24.1%	22.7%	23.0%	34.9%	33.7%	27.6%
II	35.6%	35.7%	38.3%	32.3%	32.5%	35.1%	29.3%	30.7%	31.1%	25.9%	26.9%	29.1%
III	38.6%	38.7%	37.9%	34.9%	34.2%	34.4%	38.8%	39.4%	39.1%	34.7%	34.7%	38.0%
IV	11.3%	10.0%	8.7%	4.9%	4.2%	3.1%	6.0%	5.5%	4.0%	2.9%	2.7%	3.0%
Preoperative chemotherapy	NA	NA	NA	NA	NA	NA	6.3%	6.4%	4.2%	7.8%	6.7%	3.3%
Preoperative radiotherapy	NA	NA	NA	NA	NA	NA	2.3%	2.3%	0.5%	3.7%	3.6%	0.8%
Operative results												
Operative duration (hr: min), median (IQR)	3:11 (2:28–4:05)	2:59 (2:17–3:52)	2:46 (2:08–3:40)	3:47 (3:05–4:40)	3:39 (2:56–4:33)	3:48 (2:58–4:45)	4:20 (3:16–5:42)	3:57 (2:58–5:15)	3:42 (2:50–4:52)	4:52 (3:52–6:13)	4:41 (3:41–5:59)	4:56 (3:55–6:20)
Blood loss (mL), median (IQR)	97 (30–241)	100 (35–250)	92 (34–226)	26 (5–75)	30 (8–80)	44 (10–100)	172 (43–430)	185 (50–425)	191 (77–392)	20 (5–79)	23 (5–80)	36 (10–110)
Conversion to open surgery^c^	NA	NA	NA	3.1%	2.5%	6.5%	NA	NA	NA	1.6%	1.9%	4.9%
Postoperative hospital stay (days), median (IQR)	15 (12–22)	15 (11–22)	17 (13–25)	12 (10–15)	11 (9–15)	14 (10–19)	18 (13–27)	18 (13–27)	19 (14–30)	15 (11–22)	14 (11–21)	16 (12–25)
Evaluation items												
Postoperative complication CD grade ≥3	6.1%	5.7%	6.5%	3.6%	3.8%	5.3%	10.5%	10.1%	9.4%	9.8%	9.6%	10.6%
Reoperation within 30 days	3.0%	2.9%	2.8%	2.1%	2.2%	2.5%	6.5%	6.9%	6.9%	6.3%	6.7%	7.2%
30‐day mortality	0.7%	0.8%	1.0%	0.2%	0.3%	0.2%	0.4%	0.5%	0.5%	0.2%	0.3%	0.4%

Abbreviations: ASA PS, American Society of Anesthesiologists physical status; BMI, body mass index; CD, Clavien–Dindo; COPD, chronic obstructive pulmonary disease; IQR, interquartile range; MIS, minimally invasive surgery.

^a^ This percentage is of the total procedures for each surgery type.

^b^ The proportion of robotic surgeries performed within the MIS group after 2018, when the National Clinical Database began registering robotic surgeries.

^c^ The conversion rate to open surgery pertains only to cases from 2016 to 2019.

For LAR, the percentage of MIS cases was highest in regions with ≥4 hospitals (68.0%), followed by regions with 1–3 (58.5%) hospitals and those with no hospitals (41.3%). The proportion of robotic surgery within the MIS group was highest in regions with ≥4 hospitals (8.9%). The conversion rate to open surgery was highest in the regions with no hospitals (4.9%), followed by those with ≥4 hospitals (1.6%) and 1–3 hospitals (1.9%). In both the open surgery and MIS groups, the rates for preoperative radiotherapy and chemotherapy were higher in regions with ≥4 and 1–3 hospitals than in regions with no hospitals. For both open and MIS LAR, regions with ≥4 hospitals had younger patients than regions with no hospitals. Postoperatively, for open LAR, the CD grade ≥3 complication rate was higher in regions with ≥4 hospitals (10.5%) than in those with no hospitals (9.4%). However, for MIS LAR, the complications rate was lower in regions with ≥4 hospitals (9.8%) than those with no hospitals (10.6%). The 30‐day mortality rate was lower in regions with ≥4 hospitals (0.2%) than in regions with no hospitals (0.4%).

### 3.4 Income

The results stratified by income are summarized in Table 4. For RHC, the proportion of MIS cases was higher in the highest income quartile than in the lowest income quartile (50.3% vs. 44.3%). Most residents in the highest income quartile were from urban areas (95.4% in the open surgery group and 96.3% in the MIS group). Conversely, in the lowest income quartile, a higher percentage of residents were from rural areas (30.1% and 22.4% in the open surgery and MIS groups, respectively). Based on income, no differences in treatment outcomes were observed neither in the open surgery nor in the MIS groups.

**TABLE 4:** Income.

	Right hemicolectomy								Low anterior resection							
	Open surgery				MIS				Open surgery				MIS			
	First quartile (*N* = 14 724)	Second quartile (*N* = 14 766)	Third quartile (*N* = 16 441)	Fourth quartile (*N* = 18 514)	First quartile (*N* = 14 872)	Second quartile (*N* = 15 119)	Third quartile (*N* = 14 611)	Fourth quartile (*N* = 14 724)	First quartile (*N* = 11 891)	Second quartile (*N* = 11 784)	Third quartile (*N* = 12 857)	Fourth quartile (*N* = 13 431)	First quartile (*N* = 20 441)	Second quartile (*N* = 21 748)	Third quartile (*N* = 18 761)	Fourth quartile (*N* = 16 052)
Income per year (in 1000 yen), median (range)	3750 (3405–6343)	3153 (3016–3398)	2794 (2695–3013)	2486 (1886–2691)	3766 (3405–6343)	3153 (3016–3398)	2794 (2695–3013)	2545 (1886–2691)	3750 (3405–6343)	3153 (3047–3398)	2794 (2695–3013)	2486 (1886–2691)	3766 (3405–6343)	3153 (3016–3398)	2794 (2695–3013)	2525 (1886–2691)
Urban areas, cases (%)	14 042 (95.4%)	9425 (63.8%)	3124 (19.0%)	0 (0%)	14 326 (96.3%)	10 371 (68.6%)	4508 (30.9%)	0 (0%)	11 228 (94.4%)	7504 (63.7%)	2345 (18.2%)	0 (0%)	19 673 (96.2%)	14 220 (65.4%)	5711 (30.4%)	0 (0%)
Regional city areas, cases (%)	649 (4.4%)	5341 (36.22%)	12 478 (75.9%)	12 942 (69.9%)	543 (3.7%)	4748 (31.4%)	9586 (65.6%)	11 425 (77.6%)	657 (5.5%)	7280 (36.3%)	9926 (77.2%)	9604 (71.5%)	768 (3.8%)	7528 (34.6%)	12 540 (66.8%)	12 689 (79.0%)
Rural areas, cases (%)	33 (0.2%)	0 (0%)	839 (5.1%)	5572 (30.1%)	3 (0.002%)	0 (0%)	517 (3.5%)	3299 (22.4%)	6 (0.1%)	0 (0%)	586 (4.6%)	3827 (28.5%)	0 (0%)	0 (0%)	510 (2.7%)	3363 (21.0%)
Percentage of MIS implementation^a^	NA	NA	NA	NA	50.3%	50.6%	47.1%	44.3%	NA	NA	NA	NA	63.2%	64.9%	59.3%	54.4%
The proportion of robotic surgery^b^	NA	NA	NA	NA	0.2%	0.2%	0.2%	0.2%	NA	NA	NA	NA	8.1%	8.0%	5.1%	2.8%
Patient characteristics																
Age, years, median (IQR)	75 (68–81)	75 (68–81)	76 (68–82)	76 (68–83)	74 (67–80)	74 (67–80)	74 (67–80)	74 (67–81)	68 (61–75)	69 (62–76)	69 (62–76)	69 (62–77)	67 (59–74)	68 (60–74)	68 (61–75)	68 (61–75)
Sex, male	49.6%	51.0%	48.8%	47.2%	51.0%	50.2%	49.9%	49.2%	65.0%	65.6%	64.0%	65.4%	65.3%	65.6%	64.7%	66.0%
BMI (kg/m^2^)	21.5 (19.2–24.0)	21.5 (19.3–24.0)	21.7 (19.4–24.1)	21.7 (19.4–24.2)	22.3 (20.1–24.7)	22.4 (20.1–24.7)	22.5 (20.3–24.8)	22.6 (20.3–24.9)	22.0 (19.7–24.3)	22.0 (19.7–24.4)	22.2 (20.0–24.5)	22.1 (19.9–24.5)	22.4 (20.2–24.8)	22.5 (20.3–24.8)	22.5 (20.3–24.9)	22.6 (20.4–24.9)
ASA PS 2≤	77.6%	79.3%	79.3%	81.2%	80.4%	82.6%	81.1%	83.7%	69.2%	70.7%	69.1%	72.1%	71.8%	75.4%	74.0%	76.2%
Diabetes mellitus	18.3%	19.8%	20.2%	20.3%	19.9%	20.9%	21.1%	20.8%	17.1%	18.9%	19.2%	19.3%	17.1%	18.4%	18.9%	19.2%
COPD	3.2%	3.6%	2.9%	2.8%	4.0%	3.5%	2.8%	2.7%	3.4%	4.1%	3.0%	3.3%	4.2%	3.7%	3.2%	3.2%
Cardiovascular disease	39.3%	42.3%	44.6%	48.0%	43.3%	45.5%	46.5%	50.1%	34.4%	36.8%	38.4%	40.4%	34.8%	39.0%	40.1%	42.2%
Hemodialysis	0.7%	0.9%	0.8%	0.7%	0.8%	0.7%	0.8%	0.7%	0.4%	0.5%	0.6%	0.5%	0.4%	0.5%	0.5%	0.6%
Tumor stage																
0	2.2%	1.7%	1.8%	1.9%	3.3%	3.0%	3.8%	3.4%	1.6%	1.6%	1.8%	1.9%	1.8%	1.7%	2.2%	2.2%
I	14.7%	12.9%	12.7%	13.6%	27.0%	23.9%	24.9%	25.3%	23.3%	22.2%	23.2%	23.2%	35.4%	33.1%	33.7%	33.8%
II	35.5%	36.1%	36.0%	35.6%	32.3%	33.0%	32.7%	32.2%	30.0%	31.3%	30.6%	29.9%	27.2%	26.7%	26.1%	26.3%
III	37.8%	38.9%	38.5%	39.1%	33.1%	35.3%	34.2%	35.0%	39.4%	39.1%	38.8%	39.7%	33.1%	35.7%	35.3%	34.9%
IV	9.8%	10.3%	11.0%	9.7%	4.3%	4.7%	4.4%	4.1%	5.7%	5.8%	5.5%	5.3%	2.6%	2.8%	2.7%	2.8%
Preoperative chemotherapy	NA	NA	NA	NA	NA	NA	NA	NA	7.7%	5.3%	5.7%	6.6%	8.7%	6.5%	6.0%	6.3%
Preoperative radiotherapy	NA	NA	NA	NA	NA	NA	NA	NA	3.6%	1.7%	2.1%	1.5%	5.3%	3.1%	2.6%	3.0%
Operative results																
Operative duration (hr: min), median (IQR)	3:05 (2:21–3:58)	3:02 (2:21–3:55)	2:57 (2:15–3:50)	3:01 (2:19–3:56)	3:40 (2:57–4:32)	3:45 (3:03–4:39)	3:41 (2:58–4:35)	3:41 (2:57–4:38)	4:07 (3:06–5:29)	4:03 (3:03–5:22)	3:53 (2:51–5:09)	4:03 (3:03–5:24)	4:43 (3:44–6:03)	4:46 (3:47–6:07)	4:42 (3:42–5:56)	4:46 (3:45–6:08)
Blood loss (mL), median (IQR)	100 (30–260)	100 (40–253)	100 (36–244)	90 (30–226)	30 (5–80)	30 (5–80)	30 (10–80)	30 (9–80)	184 (50–450)	190 (50–440)	176 (50–410)	185 (58–408)	22 (5–80)	20 (5–75)	22 (5–78)	25 (5–90)
Conversion to open surgery^c^	NA	NA	NA	NA	2.1%	2.9%	3.2%	2.8%	NA	NA	NA	NA	1.6%	1.6%	2.0%	2.1%
Postoperative hospital stay (days), median (IQR)	14 (11–21)	15 (11–22)	16 (12–23)	16 (12–23)	11 (9–14)	11 (9–15)	12 (10–16)	12 (9–16)	17 (13–26)	17 (13–27)	18 (13–27)	19 (14–30)	14 (11–20)	14 (11–21)	15 (12–22)	15 (11–23)
Evaluation items																
Postoperative complication CD grade ≥3	5.7%	6.1%	5.6%	6.0%	4.0%	3.7%	3.5%	3.8%	10.2%	10.3%	9.7%	10.3%	9.8%	9.8%	9.3%	9.9%
Reoperation within 30 days	2.9%	3.0%	2.9%	2.9%	2.1%	2.2%	2.1%	2.2%	6.2%	6.9%	6.8%	7.3%	6.0%	6.6%	6.5%	7.4%
30‐day mortality	0.7%	0.8%	0.7%	0.8%	0.2%	0.2%	0.2%	0.3%	0.4%	0.6%	0.4%	0.4%	0.1%	0.2%	0.3%	0.3%

*Note*: First quartile is the highest and fourth quartile is the lowest.

Abbreviations: ASA PS, American Society of Anesthesiologists physical status; BMI, body mass index; CD, Clavien–Dindo; COPD, chronic obstructive pulmonary disease; IQR, interquartile range; MIS, minimally invasive surgery.

^a^ This percentage is of the total procedures for each surgery type.

^b^ The proportion of robotic surgeries performed within the MIS group after 2018, when the National Clinical Database began registering robotic surgeries.

^c^ The conversion rate to open surgery pertains only to cases from 2016 to 2019.

For LAR, the percentage of MIS cases was higher in the highest income quartile than in the lowest income quartile (63.2% vs. 54.4%). Most residents in the highest income quartile were from urban areas (94.4% and 96.2% in the open surgery and MIS groups, respectively). Conversely, in the lowest income quartile, a higher percentage of residents were from rural areas (28.5% in the open surgery group and 21.0% in the MIS group). The reoperation rate was lower in the highest income quartile than in the lowest income quartile for both open (6.2% vs. 7.3%) and MIS cases (6.0% vs. 7.4%), whereas only for MIS cases, the mortality rate was lower in the highest income quartile (0.1% vs. 0.3%).

### 3.5 Travel distance to hospital

The results based on travel distance are detailed in Table 5. For RHC, the proportion of MIS cases was higher in the longest travel distance quartile than in the shortest quartile (51.8% vs. 45.4%). No obvious differences in postoperative outcomes were observed for open cases; however, in MIS cases, the longest travel distance quartile had a lower incidence of complications (3.5% vs. 4.0%) and reoperation rate (1.9% vs. 2.2%) than the shortest quartile.

**TABLE 5:** Travel distance.

	Right hemicolectomy								Low anterior resection							
	Open surgery				MIS				Open surgery				MIS			
	First quartile (*N* = 17 998)	Second quartile (*N* = 17 034)	Third quartile (*N* = 15 862)	Fourth quartile (*N* = 13 551)	First quartile (*N* = 14 951)	Second quartile (*N* = 15 057)	Third quartile (*N* = 14 772)	Fourth quartile (*N* = 14 546)	First quartile (*N* = 12 778)	Second quartile (*N* = 12 388)	Third quartile (*N* = 12 527)	Fourth quartile (*N* = 12 270)	First quartile (*N* = 16 957)	Second quartile (*N* = 18 208)	Third quartile (*N* = 19 520)	Fourth quartile (*N* = 22 317)
Travel distance (km), median (range)	1.6 (0–2.7)	3.9 (2.7–5.5)	7.8 (5.5–12.1)	20.5 (12.1–1557.5)	1.7 (0–2.7)	3.9 (2.7–5.5)	7.8 (5.5–12.1)	22.6 (12.1–1474.5)	1.6 (0–2.7)	3.9 (2.7–5.5)	8.0 (5.5–12.1)	21.4 (12.1–1316.6)	1.7 (0–2.7)	3.9 (2.7–5.5)	7.9 (5.5–12.1)	23.6 (12.1–1337.3)
Travel time (min), median (range)	4.5 (3.0–6.1)	9.7 (7.9–11.7)	16.1 (13.4–19.1)	31.6 (24.5–44.2)	4.8 (3.3–6.4)	10.1 (8.3–12.1)	16.8 (14.0–20.1)	33.9 (26.1–48.0)	4.6 (3.1–6.1)	9.7 (7.9–11.7)	16.3 (13.6–19.5)	33.1 (25.3–46.6)	4.8 (3.3–6.3)	10.1 (8.4–12.1)	16.9 (14.2–20.2)	35.5 (27.0–51.0)
Percentage of MIS implementation^a^	NA	NA	NA	NA	45.4%	46.9%	48.2%	51.8%	NA	NA	NA	NA	57.0%	59.5%	60.9%	64.5%
The proportion of robotic surgery^b^	NA	NA	NA	NA	0.2%	0.2%	0.2%	0.2%	NA	NA	NA	NA	4.0%	5.4%	6.2%	8.7%
Patient characteristics																
Age, years, median (IQR)	76 (69–82)	76 (68–82)	75 (68–82)	75 (67–82)	75 (68–81)	74 (68–80)	74 (67–80)	73 (66–79)	70 (63–77)	69 (62–76)	69 (62–76)	67 (60–75)	69 (62–76)	68 (61–75)	67 (60–74)	66 (58–73)
Sex, male	47.9%	49.6%	49.7%	49.1%	49.2%	49.9%	50.6%	50.6%	64.6%	64.6%	64.8%	66.0%	65.4%	65.4%	65.3%	65.3%
BMI (kg/m^2^)	21.5 (19.2–23.9)	21.6 (19.2–24.0)	21.6 (19.4–24.1)	21.8 (19.5–24.2)	22.3 (20.0–24.7)	22.4 (20.2–24.7)	22.5 (20.2–24.9)	22.6 (20.3–24.9)	21.9 (19.7–24.3)	22.1 (19.8–24.5)	22.2 (19.9–24.5)	22.2 (20.0–24.5)	22.3 (20.2–24.6)	22.5 (20.2–24.8)	22.5 (20.4–24.9)	22.6 (20.5–24.9)
ASA PS 2≤	78.8%	79.5%	80.1%	79.5%	82.4%	82.5%	81.7%	81.1%	70.8%	71.3%	69.7%	69.5%	76.1%	75.5%	74.1%	72.0%
Diabetes mellitus	19.2%	19.7%	20.5%	19.3%	20.8%	20.7%	21.1%	20.1%	19.0%	19.1%	18.7%	17.8%	18.6%	19.2%	18.6%	17.2%
COPD	3.0%	3.3%	3.1%	3.0%	2.7%	3.1%	3.3%	4.0%	3.2%	3.2%	3.9%	3.4%	3.2%	3.3%	3.7%	4.1%
Cardiovascular disease	43.6%	43.9%	44.3%	43.5%	47.2%	46.8%	46.1%	45.2%	38.5%	38.4%	37.3%	36.2%	39.8%	39.7%	38.7%	37.4%
Hemodialysis	0.6%	0.8%	0.8%	0.8%	0.6%	0.8%	0.9%	0.7%	0.4%	0.7%	0.6%	0.4%	0.5%	0.6%	0.5%	0.5%
Tumor stage																
0	1.8%	1.9%	1.9%	1.9%	3.2%	3.3%	3.6%	3.4%	1.7%	1.8%	1.8%	1.6%	2.1%	2.0%	2.0%	1.7%
I	13.2%	12.9%	13.6%	14.5%	23.6%	24.5%	25.8%	27.4%	21.6%	22.4%	23.6%	24.4%	32.1%	33.0%	33.8%	36.4%
II	36.5%	36.2%	35.5%	34.6%	34.5%	33.0%	32.0%	30.5%	32.2%	30.4%	30.3%	29.0%	27.6%	27.5%	25.9%	25.8%
III	38.6%	38.9%	38.7%	38.2%	34.3%	35.0%	34.3%	34.1%	39.3%	39.6%	39.1%	39.1%	35.5%	34.8%	35.4%	33.5%
IV	9.9%	10.1%	10.2%	10.8%	4.4%	4.3%	4.4%	4.5%	5.2%	5.9%	5.3%	5.9%	2.6%	2.7%	2.9%	2.6%
Preoperative chemotherapy	NA	NA	NA	NA	NA	NA	NA	NA	5.3%	5.5%	6.6%	7.9%	5.4%	5.9%	6.7%	9.1%
Preoperative radiotherapy	NA	NA	NA	NA	NA	NA	NA	NA	1.7%	1.7%	2.4%	3.0%	2.5%	2.8%	3.1%	5.3%
Operative results																
Operative duration (hr: min), median (IQR)	2:56 (2:15–3:50)	3:00 (2:19–3:54)	3:02 (2:19–3:53)	3:07 (2:25–4:03)	3:42 (2:59–4:37)	3:42 (2:59–4:35)	3:41 (2:57–4:37)	3:42 (2:58–4:35)	3:55 (2:56–5:12)	3:59 (3:00–5:16)	4:00 (3:01–5:20)	4:11 (3:08–5:36)	4:48 (3:47–6:04)	4:45 (3:45–6:04)	4:43 (3:43–6:00)	4:43 (3:44–6:06)
Blood loss (mL), median (IQR)	100 (12–23)	100 (36–245)	100 (35–250)	98 (30–250)	30 (8–90)	30 (8–80)	30 (7–80)	25 (5–70)	187 (51–419)	182 (50–428)	183 (50–423)	180 (50–437)	30 (5–100)	23 (5–82)	20 (5–80)	20 (5–70)
Conversion to open surgery^c^	NA	NA	NA	NA	3.0%	2.7%	2.8%	2.4%	NA	NA	NA	NA	2.8%	1.9%	1.7%	1.2%
Postoperative hospital stay (days), median (IQR)	16 (12–23)	15 (12–22)	15 (11–22)	15 (11–21)	12 (9–16)	12 (9–15)	11 (9–15)	11 (9–15)	18 (13–29)	18 (13–27)	17 (13–27)	18 (13–27)	15 (11–23)	15 (11–22)	14 (11–21)	14 (11–21)
Evaluation items																
Postoperative complication CD grade ≥3	5.8%	6.0%	5.8%	5.7%	4.0%	3.7%	3.8%	3.5%	10.3%	9.5%	10.5%	10.3%	10.2%	9.5%	10.1%	9.2%
Reoperation within 30 days	2.8%	2.9%	3.2%	2.7%	2.2%	2.3%	2.1%	1.9%	7.0%	6.7%	7.1%	6.4%	7.1%	6.6%	6.8%	5.9%
30‐day mortality	0.8%	0.9%	0.7%	0.6%	0.3%	0.2%	0.3%	0.2%	0.5%	0.5%	0.6%	0.3%	0.3%	0.3%	0.2%	0.2%

*Note*: First quartile is the shortest and fourth quartile is the longest.

Abbreviations: ASA PS, American Society of Anesthesiologists physical status; BMI, body mass index; CD, Clavien–Dindo; COPD, chronic obstructive pulmonary disease; IQR, interquartile range; MIS, minimally invasive surgery.

^a^ This percentage is of the total procedures for each surgery type.

^b^ The proportion of robotic surgeries performed within the MIS group after 2018, when the National Clinical Database began registering robotic surgeries.

^c^ The conversion rate to open surgery pertains only to cases from 2016 to 2019.

For LAR, the proportion of MIS cases was higher in the longest travel distance quartile than in the shortest quartile (64.5% vs. 57.0%). In open surgery cases, the shortest travel time quartile had a slightly higher mortality rate than the longest quartile (0.5% vs. 0.3%), whereas in MIS cases, the shortest travel distance quartile had a higher incidence of postoperative complications (10.2% vs. 9.2%) and reoperation rate (7.1% vs. 5.9%) than the longest quartile.

## 4 DISCUSSION

This study revealed that regional, economic, and patient access pattern differences may have led to variations in the penetration rates and treatment outcomes of MIS for colorectal cancer. Within the MIS LAR group, regional differences were also found in the proportion of robotic surgeries performed. MIS has been shown to be safe and non‐inferior to open surgery for the treatment of colorectal cancer and to benefit patients owing to its less invasive nature. However, as revealed in this study, previous reports demonstrating these outcomes may have primarily reflected data from facilities with better treatment outcomes, suggesting that there may be room for improvement in treatment outcomes nationwide.

In terms of regional differences, MIS treatment outcomes were better in urban areas with numerous designated cancer care hospitals, which facilitate the implementation of advanced medical care. Although in this study regions with fewer physicians did not have poor outcomes, previous reports have shown that urban areas are correlated with a greater number of physicians.^13^, ^14^ Furthermore, regions with numerous designated cancer care hospitals are concentrated in urban areas.^10^ Consequently, young doctors seeking training opportunities gather in urban areas to improve their MIS skills through interactions with physicians and participation in academic activities. Japan's Endoscopic Surgical Skill Qualification System has produced many certified professionals, contributing significantly to improving MIS treatment outcomes.^15^, ^16^ However, most certified professionals are concentrated in urban areas, although their distribution in each secondary medical region was not investigated in detail in this study. This study identified that these potential regional differences between urban and non‐urban areas may lead to disparities in MIS patient outcomes, and these regional disparities need to be addressed. In particular, advances in tele‐health and communications will be crucial for bridging these regional gaps. For example, a system for direct collaboration with certified professionals and robotic surgery instructors should be created between neighboring secondary medical regions; furthermore, a remote medical system with two‐way communication should be established between urban and remote surgeons using high‐speed communication.^17^, ^18^ Additionally, policies to encourage physicians skilled in advanced medical care to work in nonurban areas should be considered. Cancer treatment requires not only a single surgery but also long‐term postoperative care. While centralizing surgeries in high‐volume centers may be a potential solution, it risks concentrating physicians in these urban areas, exacerbating physician maldistribution. Therefore, establishing regional hub hospitals is essential to ensure equitable physician distribution and reduce patients' long‐term travel burdens. Remote medical support may play a critical role in this approach.

Although a detailed investigation into the distribution of radiation and medical oncologists has not been conducted, the higher rates of preoperative radiotherapy and chemotherapy observed in urban areas, regions with numerous designated cancer care hospitals, and regions with more physicians suggest potential regional disparities in their availability. Despite potential increases in surgical difficulty after preoperative therapy, our study identified better treatment outcomes in regions where preoperative therapies are more frequently performed. This indicates that considerable regional disparities may exist in outcomes for high‐risk or complex cases.

This study also examined the economic status and access patterns of patients. Notably, patients residing in high‐income areas had higher MIS penetration rates and better treatment outcomes. Higher income is known to correlate with better prognosis for many cancer types, including colorectal cancer.^19^, ^20^ In Japan, the universal healthcare system ensures access to medical institutions regardless of income.^21^ However, this analysis revealed that higher income was also correlated with better short‐term treatment outcomes. Income remains a factor related to hospital accessibility, and higher‐income individuals generally demonstrate better compliance with medical visits.^22^ Moreover, high‐income patients are more likely to choose to visit hospitals with better MIS treatment outcomes, despite increases in the associated travel burdens. Notably, patients in higher income groups were predominantly urban residents, whereas those in lower income groups were more likely to reside in rural areas. This suggests that patients in higher income groups may have accessed advanced facilities in urban areas due to proximity rather than deliberate effort.

The analysis of travel distance showed that the proportion of MIS cases was higher in the longer travel distance group, suggesting that these patients were willing to travel to advanced facilities that offered better MIS outcomes. Many of these advanced facilities are located in urban areas, regions with many physicians, or regions with many designated cancer care hospitals. In contrast, in Western countries and particularly in the United States, patients with longer travel distances and a greater burden of medical visits are more likely to be diagnosed with advanced stages of cancer and have poorer treatment outcomes.^23^ Japan's relatively small land area may allow patients to undergo surgery within or outside their secondary medical regions, despite the distance, leading to better surgical outcomes. However, the number of hospitals in Japan is expected to decrease in the future owing to population decline.^24^ As a result, similar to what is seen in Western countries, there is a risk that patients facing greater burdens will have difficulties accessing hospitals to maintain good outcomes. Thus, in addition to policies for centralizing hospitals that perform highly complex surgeries,^25^ establishing systems to reduce regional disparities is essential to prepare for this eventuality.

In previous NCD studies, the 30‐day mortality rate for right hemicolectomy was reported to be 1.1%–1.3%, which is higher than the 0.3%–0.4% observed in low anterior resection, suggesting a greater complexity for right hemicolectomy.^6^ However, in the present study, the mortality rate for right hemicolectomy was 0.2%–0.4%, which is lower than previously reported. Unlike earlier reports, we excluded cases involving emergency surgeries and simultaneous surgeries in other regions; we believe that this contributed to the disparity found between our results and those of previous studies.

This study revealed regional differences in the proportion of robotic surgeries for rectal cancer within the MIS group. In contrast, robotic surgeries for colon cancer were not covered by Japan's insurance system until 2022; consequently, they were performed in a limited number of facilities during the study period, making it difficult to assess regional disparities. However, now that insurance coverage has been granted, regional differences in robotic surgeries for colon cancer may become more apparent, similar to those observed for rectal cancer.

Notably, when comparing open surgery and MIS within the same procedure and region, BMI and cardiovascular complication rates were consistently higher in the MIS group than in the open surgery group. The results of this analysis suggest that regional disparities exist in MIS utilization and that MIS tends to be performed more frequently in high‐BMI cases. BMI and cardiovascular complications are interrelated factors, and this tendency to favor MIS over open surgery in obese patients is likely due to its perceived benefits. Furthermore, the conversion rate to open surgery was found to be lower in urban areas, regions with numerous designated cancer care hospitals, and regions with more physicians than in rural areas, regions with no designated hospitals, and regions with few physicians, respectively. This implies that urban areas and regions with numerous designated cancer care hospitals and more physicians can actively perform MIS for challenging cases while maintaining high‐quality outcomes for MIS procedures. Consequently, MIS for particularly challenging cases may be a promising target for remote guidance or consultation initiatives.

This study had some limitations. First, our results may not reflect the current situation because we targeted cases from 2013 to 2019. Second, the study focused on colorectal cancer; whether the same trends apply to other cancer types remains unclear. Third, the complex interactions between treatment outcomes and factors such as patient background, including age, PS, BMI, comorbidities, tumor stage, surgery duration, blood loss, and differences in surgical skill were not evaluated. Fourth, although ArcGIS can calculate travel time by foot or bike, we evaluated using cars as a universally available option. However, in urban areas with advanced transit systems, public transport usage may impact our results. Lastly, although this study demonstrated differences in outcomes, these are not substantial enough to definitively assert a causal relationship between outcomes and regional disparities.

In conclusion, this analysis using high‐quality NCD data for colorectal cancer procedures subject to medical standard evaluation revealed disparities in MIS treatment outcomes when stratifying by characteristics of secondary medical regions and patients' economic status. In future medical policies, implementing measures to facilitate information sharing and the collaboration of urban regions (particularly those with many physicians and designated cancer care hospitals) with other regions is imperative to improve MIS treatment outcomes nationwide.

## AUTHOR CONTRIBUTIONS

**Atsushi Hamabe:** Conceptualization; data curation; formal analysis; writing – original draft. **Arata Takahashi:** Data curation; formal analysis; writing – original draft. **Hiraku Kumamaru:** Data curation; formal analysis; writing – original draft. **Hiroshi Hasegawa:** Formal analysis; writing – original draft. **Koki Otsuka:** Formal analysis; writing – original draft. **Yoshihiro Kakeji:** Formal analysis; writing – review and editing. **Ken Shirabe:** Formal analysis; writing – review and editing. **Masafumi Inomata:** Formal analysis; writing – original draft. **Yuko Kitagawa:** Formal analysis; writing – review and editing. **Ichiro Takemasa:** Conceptualization; formal analysis; writing – original draft.

## FUNDING INFORMATION

This study was supported by the Japan Society for Endoscopic Surgery (JSES).

## CONFLICT OF INTEREST STATEMENT

Yoshihiro Kakeji, Ken Shirabe, Masafumi Inomata, Yuko Kitagawa, and Ichiro Takemasa are editorial members of *Annals of Gastroenterologial Surgery*. Arata Takahashi and Hiraku Kumamaru are affiliated with the Department of Healthcare Quality Assessment at the University of Tokyo. THe department is a social collaboration department supported by National Clinical Database, Johnson & Johnson K.K., Nipro Corporation, and Intuitive Surgical Sàrl. H.K. reports receiving consultation fee from EPS Corporation, and a research grant on an unrelated subject from Amgen KK and Pfizer KK. The other authors declare no conflicts of interest for this article.

## ETHICS STATEMENT

Approval of the research protocol by an Institutional Reviewer Board: The study protocol was approved by the institutional review board of Sapporo Medical University Hospital (3‐1‐29) and conducted in compliance with the tenets outlined in the Declaration of Helsinki.

Informed Consent: The requirement for patient consent was waived.

Registry and the Registration No. of the study/trial: N/A.

Animal Studies: N/A.

## Data Availability

Not applicable.
